# P-1036. Ventilator-Associated Pneumonia Caused by Gram-negative Microorganisms in Intensive Care Units of a Northeastern Colombian Tertiary Hospital. Case Description and Risk Factor Analysis From a Six Year Retrospective Cohort

**DOI:** 10.1093/ofid/ofaf695.1231

**Published:** 2026-01-11

**Authors:** Juan D Berlinghieri Perez, Nathalia A Rodriguez Villarreal, Claudia L Figueroa Pineda, Agustin Vega Vera, Francisco F Naranjo Junoy

**Affiliations:** Universidad Industrial de Santander, Bucaramanga, Santander, Colombia; Universidad Industrial de Santander, Bucaramanga, Santander, Colombia; Universidad Industrial de Santander, Bucaramanga, Santander, Colombia; Universidad Industrial de Santander, Bucaramanga, Santander, Colombia; Universidad Industrial de Santander, Bucaramanga, Santander, Colombia

## Abstract

**Background:**

Advances in intensive care have led to reduced mortality in many critical illnesses; however, ventilator-associated pneumonia (VAP) remains a serious complication. This study analyzes the clinical characteristics, microbiological findings, and factors associated with mortality in patients with VAP in a Colombian hospital between 2018 and 2024.

**Table 1.**
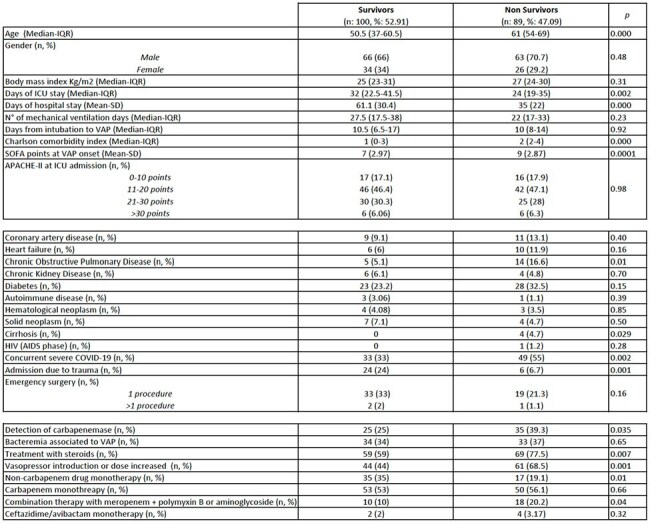
Patients general characteristics of ventilator associated pneumonia (VAP) caused by gram negative microorganisms

**Image 1.**
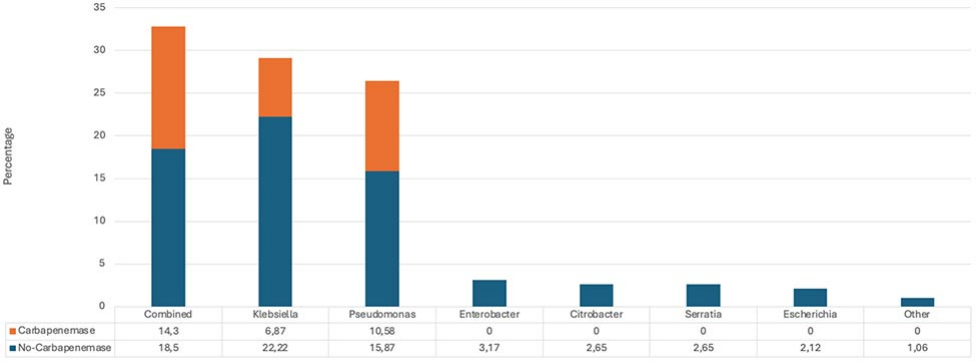
Microbiological isolates and carbapenemase expression

**Methods:**

A retrospective cohort study was conducted in a tertiary hospital in Bucaramanga, Colombia, which has 50 intensive care beds. The study included 189 patients aged over 18 years diagnosed with VAP according to CDC criteria and with bronchial secretion cultures taken within 48 hours of symptom onset. Patients with sole isolation of gram-positive pathogens were excluded from the analysis. The primary outcome was in-hospital mortality. Risk factors for mortality were evaluated by comparing variables in survivors and non-survivors.

**Image 2.**
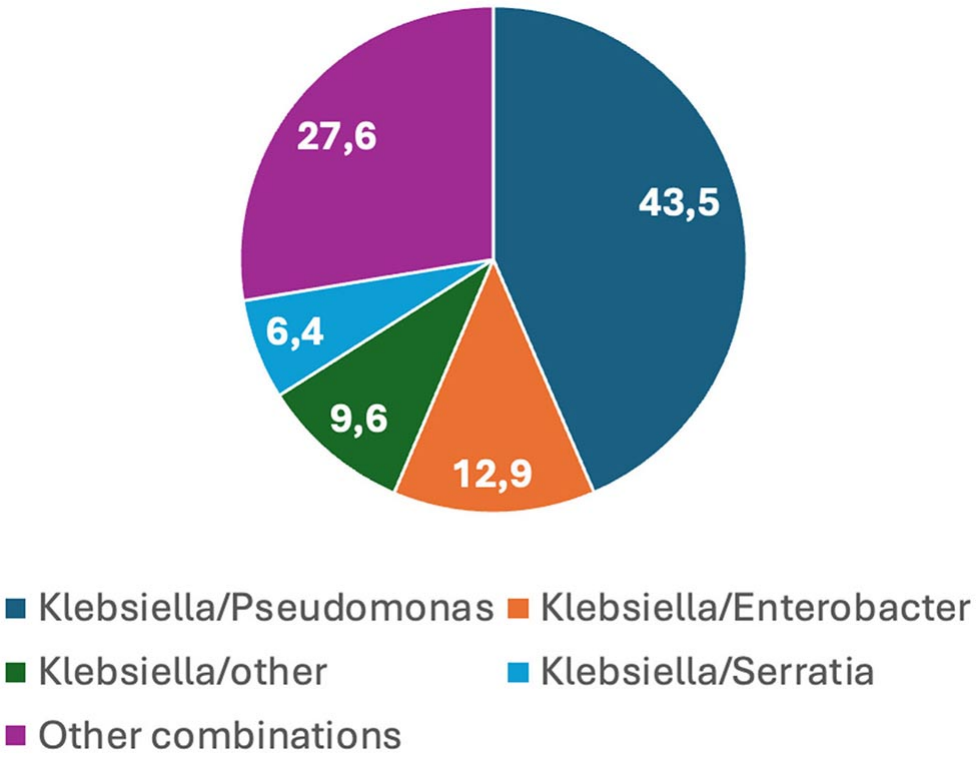
Distribution of combined isolates (percentage)

**Table 2.**
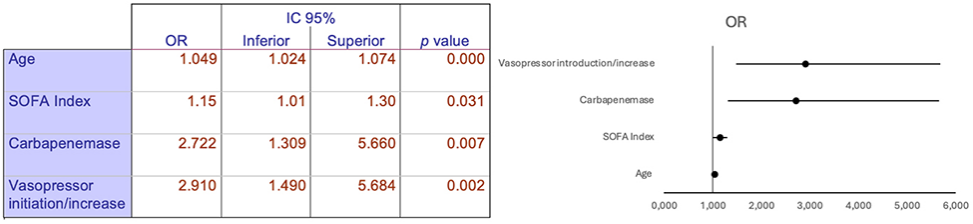
Multivariate analysis of risk factors for mortality in ventilator-associated pneumonia caused by gram negative microorganisms

**Results:**

Male gender predominated (68%), and the median age was 58 years (IQR: 46-69). The average lenght of hospital stay was 48 days (SD 29.7), and the median time from intubation to VAP onset was 10 days (IQR: 8-17). The Charlson comorbidity index was low with a median of 2 points (IQR: 0-3). 43% of patients had severe concurrent COVID-19. Overall mortality was 47%. The most frequent pathogens were *Klebsiella Sp.* (29.1%) and *Pseudomonas Sp.* (26.4%). In 32% of cases a combination of two pathogens were isolated. Carbapenemases were detected in 31.75% of cases, mainly in *Pseudomonas* (40%). Factors associated with higher mortality included advanced age, a high Sequential Organ Failure Assesment (SOFA) score at the time of VAP diagnosis, infection by a carbapenemase-producing microorganism, and the need for initiation or increase in vasopressor drugs.

**Conclusion:**

A high concurrence with severe COVID-19 was observed in our population what could influence in a higher mortality rates than previously reported. *Klebsiella* and *Pseudomonas* were the most predominant isolated genera similar to regional epidemiology. Age, degree of organ dysfunction, infection with carbapenem resistant bacteria and vasopresor requirements were all factors that were associated with higher mortality at VAP onset.

**Disclosures:**

All Authors: No reported disclosures

